# Supplementary material for: Ex Vivo Traceability Platform for Phospholipoproteomic Formulations: Functional Evidence Without Clinical Exposure
Source: Biomedicines. 2025 Aug 28;13(9):2101. doi: 10.3390/biomedicines13092101 (PMC12467205; doi:10.3390/biomedicines13092101)
Supplement: Supplementary file 1 [file biomedicines-13-02101-s001.zip › biomedicines-3716421-supplementary.pdf]

biomedicines

MDPI

Supplementary Text S1. Structure and Logic of the STIP Immunophenotypic Decision Tree

The immunophenotypic decision tree implemented in the STIP (Structured Traceability and Immunophenotypic Platform) system provides a structured and reproducible method for classifying phenotypic responses to phospholipoproteomic vesicular formulations under standardized ex vivo conditions. Unlike traditional molecular approaches that rely on genomic markers or pharmacodynamic targets, the STIP model uses real-time functional variables—such as confluence divergence, non-destructive cell death signals, and secretome ratios—to determine functional compatibility. For divergence calculations, confluence values were normalized to baseline (T_0) and expressed as percentage change over time using the following formula:

ΔC_t = (C_t – C_t0)/C_t0 × 100

Structure and Node Logic

The decision tree is organized hierarchically and begins by evaluating whether there is a significant divergence compared to the untreated control. If the response curve remains within ±10% of control values for at least 12 hours and does not show statistical significance ($p > 0.05$), the response is classified as Neutral (Type III), and no further stratification is applied.

For profiles with significant divergence, the next node evaluates the direction of change:

Positive divergence (Δ confluence ≥ +20%): The tree then examines proliferation slope, plateau stability, and cytokine levels. A consistent increase in IL-6 with detectable IL-10 within permissive ranges leads to classification as **Stimulatory (Type I)**, consistent with non-inflammatory trophic integration.

Negative divergence (Δ confluence ≤ −20%): This directs the logic to the **Inhibitory branch (Type II)**. The system evaluates the cumulative non-destructive death signal and the IFN-γ / IL-10 ratio. A profile with low death (<3%), elevated IFN-γ, and suppressed IL-10 is classified as Inhibitory (Type II), consistent with immune-induced structural arrest (e.g., via p21 or GADD45 pathways).

If cell death exceeds 5% or morphology is compromised, the record is flagged as **Structurally Disruptive**, and excluded from STIP classification.

Functional Outcome

Each final branch yields a coded output (Type I, II, III), which is recorded in the STIP technical dossier along with raw and processed metrics. This logic tree ensures consistency across vesicle–cell interactions, minimizes analyst bias, and supports reproducibility in regulatory documentation.

Future Extensions

The decision tree is designed for integration with algorithmic systems such as SAP and may be adapted to immunophenotypic prediction models. This compatibility transforms STIP from a static classifier into a dynamic decision-support tool, enabling real-time logic-based validation for vesicular products.

Table S1. Summary of STIP Functional Records.

Record ID	Vesicle Lot	Sentinel Line	Δ Confluence (%)	FSI Score	IFN-γ/IL-10	Functional Category	Cross-Validation
STIP-001	FV-001	BEWO	+34.1	+42.3	2.1	Stimulatory	✓
STIP-002	FV-002	A375	−28.7	−26.1	5.9	Inhibitory	✓

 https://doi.org/10.3390/biomedicines13092101

STIP-003	FV-003	MCF-7	+1.6	+3.2	1.0	Neutral	✓
STIP-004	FV-004	BEWO	+31.8	+39.5	2.5	Stimulatory	✓
STIP-005	FV-005	A375	−29.5	−28.3	6.1	Inhibitory	✓

Functional classification and core metrics from STIP system outputs. Each row corresponds to one of the 112 functional records formally validated and included in the present publication. Data include vesicle lot ID, sentinel tumor cell line applied, magnitude of confluence divergence (Δ%), computed Functional Stratification Index (FSI), secretome ratio (IFN-γ/IL-10), and final classification into one of three immunophenotypic response categories (Stimulatory, Inhibitory, Neutral). The last column confirms cross-validation under replicates and inter-lot conditions. In total, the STIP system has been applied to over 500 technical records for documentary purposes; only the subset validated in this study is reported here.

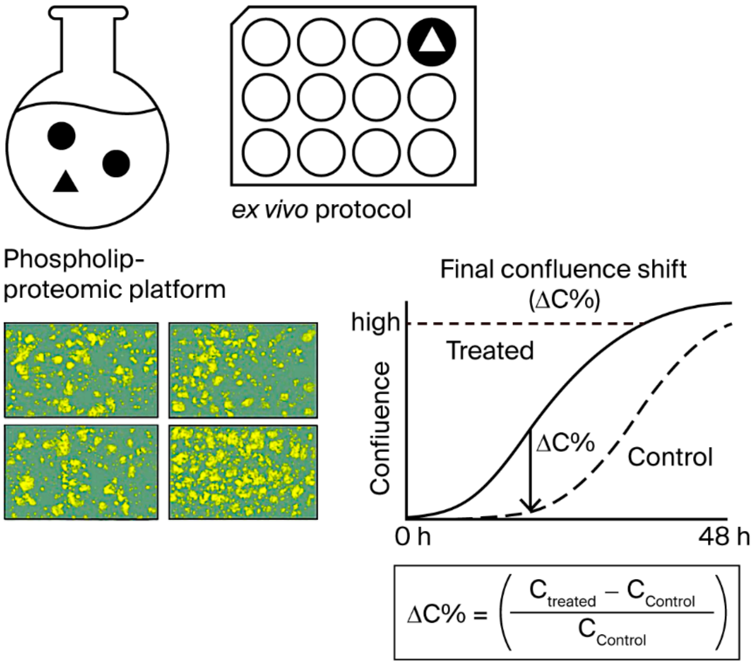

Figure S1. STIP Workflow Real-Time (ΔC%). *Assessment for Phospholipoproteomic Platforms.* This diagram provides an integrated overview of the STIP system as applied to non-pharmacodynamic phospholipoproteomic platforms. The workflow illustrates: (1) registration and preparation of the bioendogenous formulation, (2) implementation of the ex vivo protocol in multi-well plates, (3) sequential imaging of cell confluence over 48 h, and (4) derivation of the Final Confluence Shift (ΔC%) between treated and control wells, calculated through the indicated formula. Final Confluence Shift was defined as: ΔC% = (C_t48h treated − C_t48h control) / C_t48h control × 100. The process enables reproducible, label-free functional documentation without toxicity, systemic exposure, or therapeutic claims—supporting regulatory inclusion under CTD Module 5.3 and SAP documentation pathways. Images were acquired at 10× magnification (scale bar = 100 μm, applies to all panels).

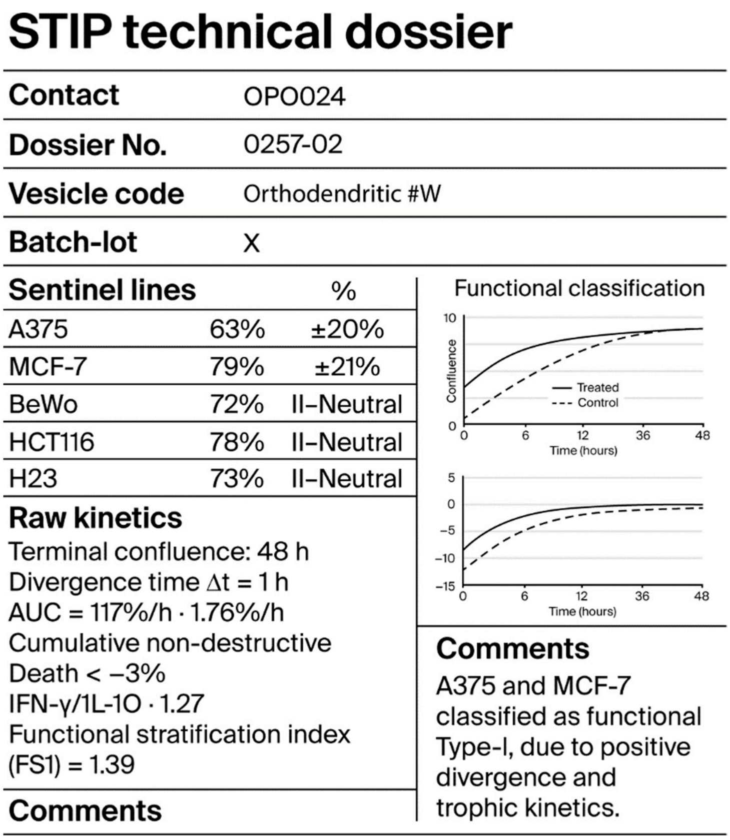

STIP technical dossier

**Contact**	OPO024
**Dossier No.**	0257-02
**Vesicle code**	Orthodendritic #W
**Batch-lot**	X

**Sentinel lines**		%
A375	63%	±20%
MCF-7	79%	±21%
BeWo	72%	II–Neutral
HCT116	78%	II–Neutral
H23	73%	II–Neutral

Raw kinetics

Terminal confluence: 48 h
Divergence time Δt = 1 h
AUC = 117%/h · 1.76%/h
Cumulative non-destructive
Death < −3%
IFN-γ/1L-1O · 1.27
Functional stratification index
(FS1) = 1.39

Comments

Comments

A375 and MCF-7 classified as functional Type-I, due to positive divergence and trophic kinetics.

Figure S2. Sample STIP Technical Dossier. Example of a fully completed STIP (Structured Traceability and Immunophenotypic Platform) technical dossier, summarizing key outputs from a standardized vesicle–sentinel run. The document includes batch identification, sentinel line responses, kinetic divergence, death signal, IFN-γ/IL-10 ratio, and final functional classification. Raw kinetic metrics are presented alongside a representative proliferation curve. This format allows cross-validation, inter-lot comparability, and traceability of phenotypic outputs under non-clinical, ex vivo conditions. Comments confirm categorical assignments and reinforce reproducibility across negative and positive controls. This document structure is used as a reference in regulatory-supporting records and STIP database archiving.

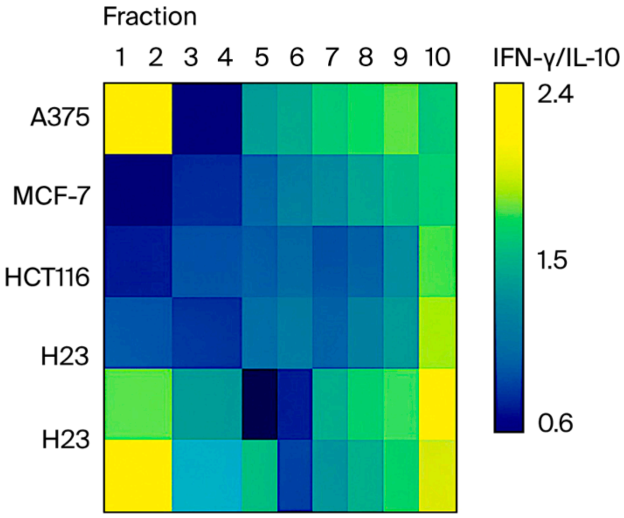

Figure S3. Inter-Lot Variability of IFN-γ/IL-10 Ratio Across Tumor Cell Lines. Heatmap illustrating secretomic variability across ten phospholipoproteomic fractions tested in five human tumor-derived cell lines (A375, MCF-7, HCT116, and H23; duplicated entries represent independent experimental runs). Color gradient reflects the magnitude of the IFN-γ / IL-10 ratio, from low values (~0.6,

dark blue) to elevated ratios (~2.4, bright yellow). Despite minor lot-to-lot fluctuations, immunophenotypic directionality remained stable across conditions, supporting the reproducibility and robustness of the STIP system for batch-level classification. These data are directly integrated into real-time STIP traceability dossiers.

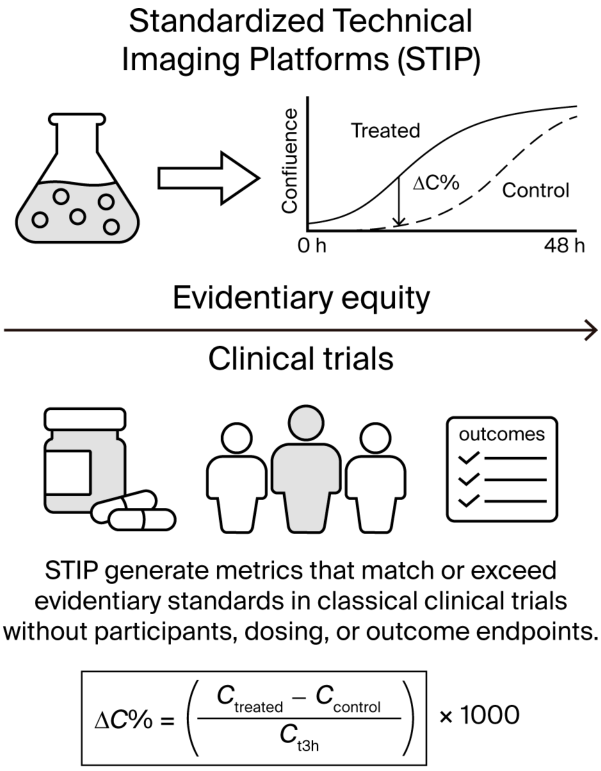

Figure S4. Comparative overview of STIP (Structured Traceability and Immunophenotypic Platform) versus classical clinical trials. The upper panel illustrates how the STIP system produces regulatory-grade documentation through real-time confluence tracking and ΔC% derivation in sentinel cell lines exposed to phospholipoproteomic platforms—without requiring patient involvement, dosing, or therapeutic endpoints. In contrast, the lower panel depicts the conventional clinical trial pathway, which depends on human participation, pharmacological administration, and predefined outcome endpoints. The arrow labeled "EVIDENTIARY EQUITY" highlights STIP's ability to match or exceed the evidentiary value of classical trials through technically standardized, reproducible, and regulatorily accepted documentation strategies.

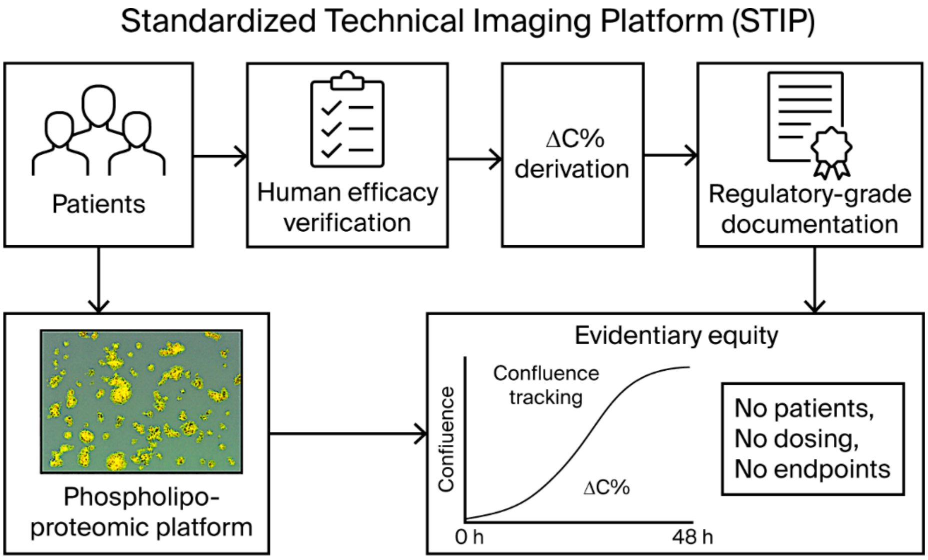

Figure S5. STIP as a Label-Free Alternative to Patient-Based Validation. Conceptual flowchart illustrating how STIP replaces patient-dependent efficacy validation with kinetic, label-free phenotypic evidence. In conventional models, clinical outcomes require human subjects, dosing protocols, and endpoint tracking. STIP bypasses this by deriving regulatory-grade metrics (e.g., ΔC%) directly from vesicle-exposed cell monolayers, enabling continuous confluence tracking over 48 h without intervention. The resulting technical output—free from intersubject variability—meets evidentiary standards for CTD 5.3 inclusion, offering evidentiary equity without clinical burden. Images were acquired at 10× magnification (scale bar = 100 μm, applies to the panel).

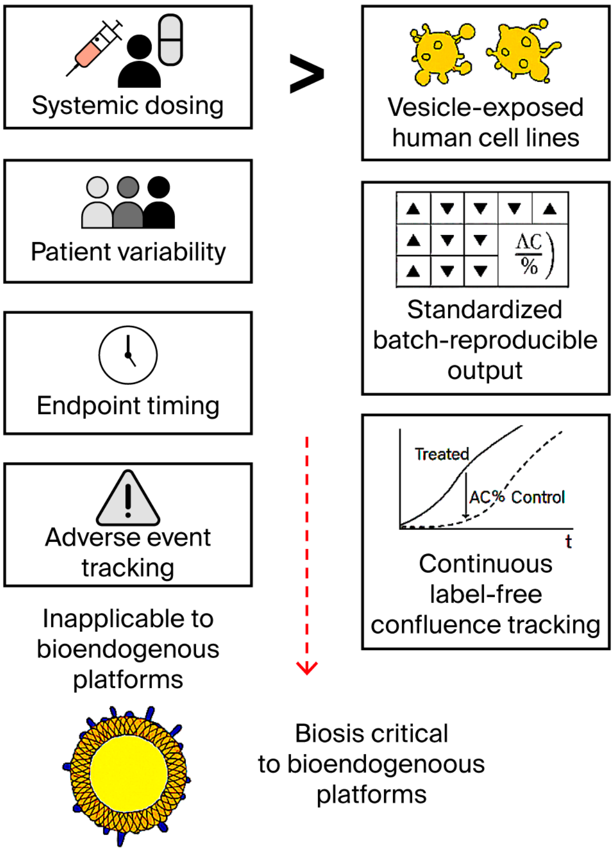

Figure S6. STIP Versus Clinical Trials for Bioendogenous Validation. This regulatory comparison illustrates why STIP (Structured Traceability and Immunophenotypic Platform) provides superior evidentiary fitness for bioendogenous products. Clinical trials rely on systemic dosing, human variability, timed endpoints, and adverse event monitoring—elements incompatible with non-pharmacodynamic vesicular platforms that lack circulation, receptor binding, or systemic toxicity. STIP replaces these requirements with batch-reproducible, real-time, label-free confluence tracking in vesicle-exposed human cell lines. It delivers ΔC%-based outputs directly from functional biosis, ensuring documentation that is not only regulatorily valid, but biologically appropriate for these structurally defined products.

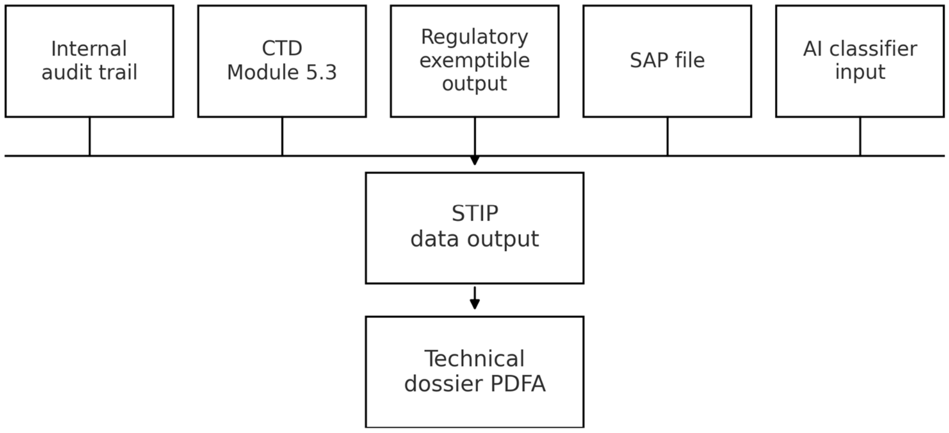

Figure S7. STIP as a Centralized Documentation Engine. STIP operates not merely as an assay but as a documentation engine: a centralized, harmonized output system that feeds directly into regulatory, institutional, and algorithmic frameworks. This diagram visualizes how STIP Data Output supports six key endpoints: CTD Module 5.3 (functional documentation), SAP file structures (preclinical audit systems), exemption justifications (e.g., for pre-IND), internal audit trails, PDF/A technical dossiers, and future-ready AI classifier inputs. Unlike clinical data, these outputs are version-controlled, reproducible, and immune to intersubject variability—positioning STIP as a scalable, regulatorily compliant evidence hub.

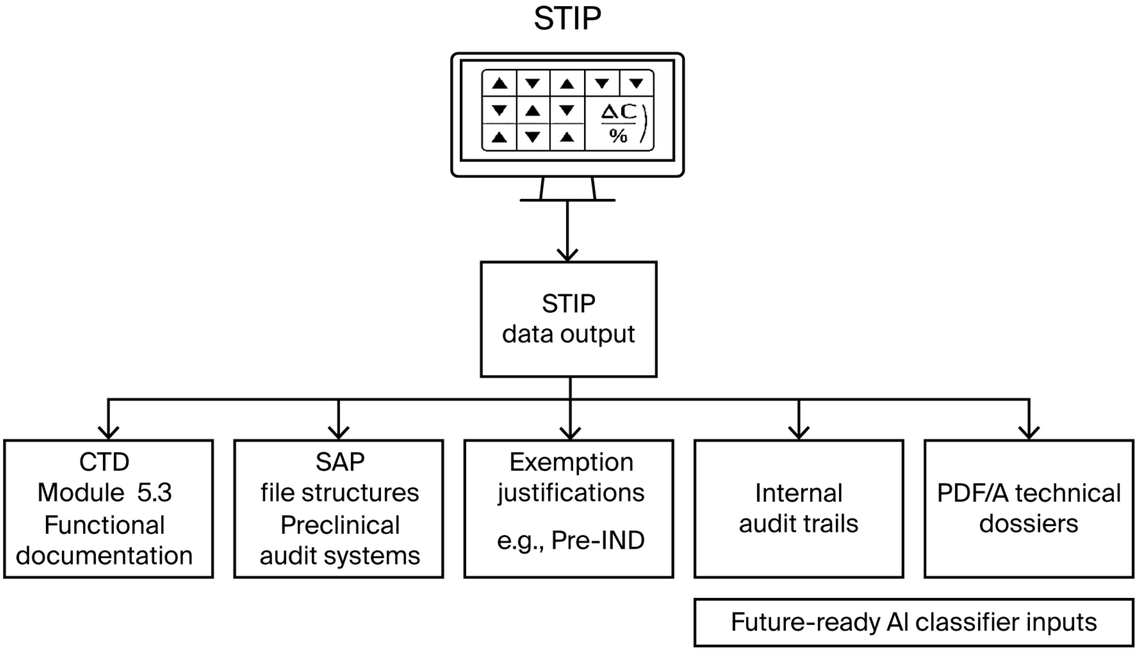

Figure S8. STIP Data Output as a Multi-Endpoint Regulatory Interface. The figure illustrates STIP (Structured Traceability and Immunophenotypic Platform) as a centralized engine for generating regulatory-grade documentation. At its core, the STIP Data Output consolidates kinetic, morphological, and secretomic data into standardized, version-controlled units. These outputs feed directly into six critical regulatory and institutional endpoints: (1) CTD Module 5.3 for non-clinical functional evidence; (2) SAP file structures used in preclinical audit systems; (3) Regulatory exemption justifications, including pre-IND dossiers; (4) Internal audit trails ensuring procedural transparency; (5) PDF/A technical dossiers for formal archiving; and (6) Future-ready AI classifier inputs that enable algorithmic stratification without clinical endpoints. Unlike traditional models that require systemic exposure or therapeutic claims, STIP produces interoperable, biosis-based evidence compatible with regulatory frameworks, clinical waivers, and institutional decision-making layers.

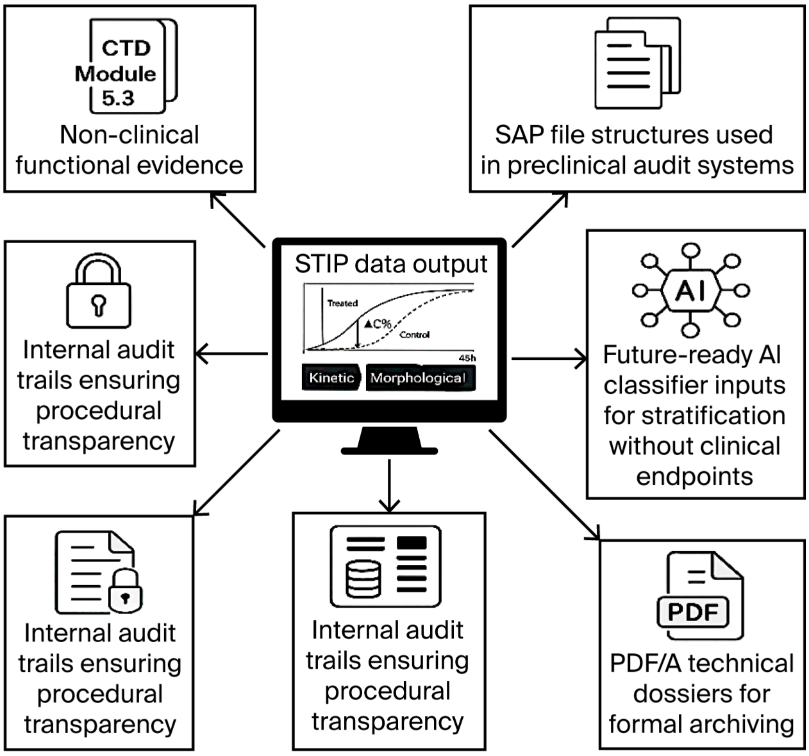

Figure S9. STIP as a Regulatory Infrastructure Scaffold. This advanced documentation diagram shows STIP as a multidimensional data engine capable of supporting six critical endpoints from a single traceable output unit. At its center, STIP Data Output merges kinetic and morphological information—including ΔC% curves—into a structured digital format. Arrows extend to (1) CTD Module 5.3 for functional validation, (2) SAP-based preclinical file structures, (3) exemption-support letters (e.g., pre-IND), (4) AI-ready classifier pipelines, (5) PDF/A-formatted regulatory dossiers, and (6) cryptographically secured audit trails. This visual emphasizes STIP's role as both an experimental protocol and a regulatory infrastructure scaffold.

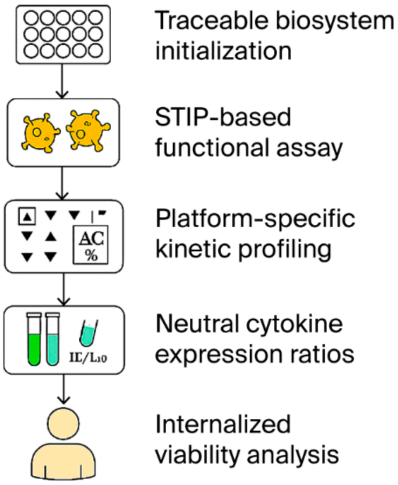

Figure S10. Regulatory Flowchart for STIP-Based Exemption Pathways. This strategic flowchart illustrates how a bioendogenous platform can achieve regulatory-grade documentation without relying on Phase 1–3 clinical trials. The process begins with traceable biosystem initialization, advances through STIP-based functional assays and kinetic profiling (including ΔC%), incorporates neutral cytokine ratios (e.g., IL-6, IL-10, IFN-γ), and concludes with viability analysis confirming structural safety. This evidence stack supports documentation frameworks that recognize exemption from clinical trials in the absence of systemic risk, pharmacodynamic action, or toxicological concern—enabling proportionate regulatory integration with deferred activation where applicable.

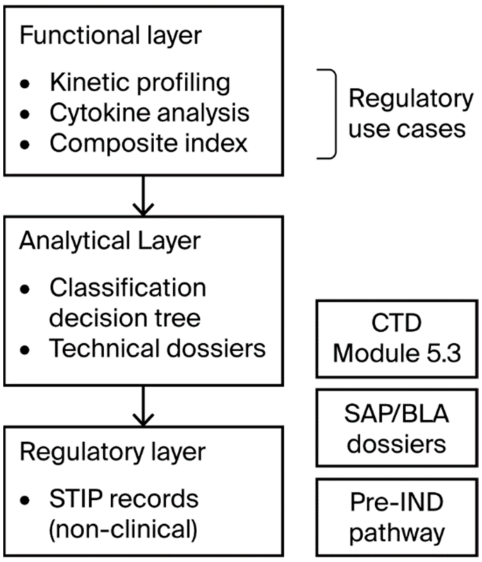

Figure S11. Scope and Technical Layers of the STIP Platform. The STIP system integrates phenotypic readouts, cytokine analysis, and kinetic profiling into a logic-based classification model, generating non-clinical technical dossiers compatible with regulatory frameworks such as CTD Module 5.3 and SAP. This visual summarizes the platform's three operational layers—functional, analytical, and regulatory—and illustrates STIP's role as a non-pharmacodynamic documentation system.

Glossary of Terms and Acronyms

The following glossary compiles the main acronyms and technical terms used in this manuscript, along with their respective operational definitions.

Acronym	Full Name	Definition/Description
**AUC**	Area Under the Curve	Cumulative metric of proliferative behavior, obtained by trapezoidal integration of the confluence curve.
**CBA**	Cytometric Bead Array	Multiplex technique for cytokine quantification in cell culture supernatants.
**CO_2**	Carbon Dioxide	Gas used to maintain physiological conditions during cell incubation.
**CRF**	Case Report Form	Internationally standardized format for the presentation of regulatory data on biological or pharmaceutical products.
**CTD**	Common Technical Document	Internationally standardized format for the presentation of regulatory data on biological or pharmaceutical products.
**DLS**	Dynamic Light Scattering	Technique for measuring particle size in vesicular fractions.
**eCRF**	Electronic Case Report Form	Digital version of the CRF; used in clinical settings to capture structured data.
**FSI**	Functional Stratification Index	Composite index calculated by weighted z-score, summarizing overall functional response in STIP.
**ΔC_t**	Delta Confluence over time	Percentage change in confluence relative to baseline (T_0), calculated as (C_t – C_t0) / C_t0 × 100.
**ΔC%**	Final Confluence Shift	Relative difference in confluence at 48 h between treated and control wells, calculated as (C_t48h treated – C_t48h control) / C_t48h control × 100.
